# Is the G-Layer a Tertiary Cell Wall?

**DOI:** 10.3389/fpls.2018.00623

**Published:** 2018-05-08

**Authors:** Bruno Clair, Annabelle Déjardin, Gilles Pilate, Tancrède Alméras

**Affiliations:** ^1^CNRS, UMR EcoFoG, AgroParisTech, Cirad, INRA, Université des Antilles, Université de Guyane, Kourou, France; ^2^BioForA, INRA, ONF, Orléans, France; ^3^LMGC, CNRS, Université de Montpellier, Montpellier, France

**Keywords:** gelatinous layer, G-layer, secondary cell wall, tertiary cell wall, tension wood, flax, maturation stress

## The G-layer: an example of plant “muscle”

Plant movements have fascinated scientists at least since Darwin's studies on this issue (Darwin and Darwin, 1880). These movements are enabled by various motor systems (Moulia et al., 2006; Forterre, 2013), and represent basic adaptations to the terrestrial environment since they are necessary to achieve adaptive movements or maintain the orientation of axes during growth (Moulia et al., 2006; Alméras and Fournier, 2009). To enable this function, a diversity of motor systems co-exists in the plant kingdom. In actively elongating organs, the mechanisms are generally based on differential growth. In non-growing soft tissues, a large variety of motor systems makes possible slow or rapid plant movements (Forterre, 2013). In woody stems, normal wood has contractile properties, named maturation strains (Archer, 1986). Combined with an eccentric secondary growth, these strains are able to induce changes in stem curvature (Alméras et al., 2005), sufficient to manage small mechanical disturbances. In the case of strong disturbances, trees generally produce reaction wood. In gymnosperms, the motor system is based on the swelling properties of a specific type of reaction wood named compression wood (Timell, 1986). In angiosperms, most species develop tension wood with fibers having a peculiar cell wall layer (Ghislain and Clair, 2017), historically named gelatinous layer or G-layer. This layer allows for the contraction of the fiber during its maturation, acting as a “muscle” to fulfill various mechanical needs, such as adjusting the shape and orientation of woody plant axes.

The underlying mechanism allowing G-layer to produce contraction, and therefore tensile stress, is not fully understood and still a matter of debate (Alméras and Clair, 2016; Gorshkova et al., 2018). The contraction occurs during cell wall thickening (Clair et al., 2011) synchronously with the swelling of the mesopores (Chang et al., 2015), suggesting a mechanism based on the swelling of the cell wall matrix that results in a strong tensile stress within the cellulosic lattice network (Alméras and Clair, 2016). Alternatively, a mechanism based on the entrapment of matrix material during cellulose aggregation has been proposed (Gorshkova et al., 2015). These two mechanisms may act together in a complementary manner (Alméras and Clair, 2016).

The G-layer was first described in 1860 by Hartig as a cellulosic, gelatinous, mucilaginous or cartilaginous layer. These names derived from its gelatinous aspect, often detached from the other layers of the fiber (after Sanio, 1860 cited by Potter, 1904). Anderson (1927) named this layer “tertiary wall” in a study on flax fibers. Wardrop and Dadswell (1948) used the term “tertiary layer” for tension wood in a single paper, but they later abandoned this term and named it G-layer. Onaka (1949) wrote the first reference monography on reaction wood, using the term G-layer, since then used by all the wood and tree community. In 1964, the International Association of Wood Anatomy Committee named this peculiar layer “G-layer” in the “Multilingual glossary of terms used in wood” (IAWA-Committee, 1964). Lately, in 2008, the G-layer was first described as having a mesoporous structure characteristic of a gel (Clair et al., 2008), providing a rational justification for the term “gelatinous.”

In 2012, Gorshkova and coworkers, working mainly on flax cortical fibers, have reintroduced the term “tertiary cell wall” in place of “G-layer,” giving more and more emphasis to this terminology in their articles (Gorshkova et al., 2012, 2015; Mellerowicz and Gorshkova, 2012; Mikshina et al., 2015), until their recent review paper entitled “Plant ‘muscles’: fibers with a tertiary cell wall” (Gorshkova et al., 2018). In the present paper, we wish to discuss the relevance to name the G-layer a “tertiary cell wall.”

## What is a “tertiary” cell wall?

Plant cell walls are often made of several layers. For example, the normal xylem fibers are made of a primary wall, and of three additional layers most often named S1, S2, and S3, usually gathered under the term “secondary cell wall.” The term “tertiary cell wall” was introduced a long time ago, but this terminology was from the beginning a matter of debate (Leise, 1963). From an ontogenic view, S1, S2, and S3 are all belonging to the secondary cell wall, whereas from a morphological view, S1 can be termed a transition layer, S2 the secondary wall, and S3 a tertiary wall (Wenzl, 1970). The IAWA glossary defined the tertiary wall as the “innermost layer of the cell wall next to the cell-lumen, often with warts” (IAWA-Committee, 1964). For some author, this “innermost layer” included the S3 layer which has a chemical composition different from the S1 and S2 layers (Leise, 1963). For this last author, the tertiary wall did not include the warty layer. The warty layer that develops during the last stages of cell differentiation, “is the remainder of the dying protoplast” (Leise, 1963). On the contrary, some authors propose to include this warty layer into the tertiary wall that is therefore made of two strata, the membranogenic stratum and the warty stratum (Frey-Wyssling, 1976). More recently, it was proposed to restrict the tertiary wall to the warty layer (Barnett and Jeronimidis, 2003). Following this last definition, the tertiary wall results from a *post-mortem* deposition and is therefore both morphologically and ontogenetically distinct from the secondary wall.

Gorshkova et al. (2018) propose that the G-layer is a tertiary cell wall with the following arguments: (i) “the G-layers are developed after the primary and secondary cell walls”, (ii) they “have distinct composition, architecture, and physical properties”, and (iii) “their formation is regulated by a set of transcription factors that differ from those of the primary and secondary cell walls.” Hereafter we discuss these arguments.

## From an ontogenic view, is the G-layer a “tertiary” cell wall?

The terms “primary” and “secondary” bear particular meaning when referring to plant development. They refer to two distinct morphogenetic phases: a phase of extension, and a phase of thickening. This distinction applies to the growth of plant organs, where primary growth refers to organ extension achieved by primary meristems and secondary growth to organ thickening achieved by secondary meristems. It also applies at the tissue scale, where primary tissues developed from the primary meristem during organ elongation and secondary tissues developed from the secondary meristems during organ thickening. Current terminology makes it consistent with the cell wall scale, where extending cells have, most of the time, only a primary cell wall, while cell wall thickens during secondary cell wall deposition. From an ontogenic view, the term “tertiary” implicitly refers to a distinct phase of development, following cell wall extension and thickening. We believe that G-layer development is part of the phase of cell wall thickening, and in consequence, it should be considered as one layer of the secondary cell wall rather than a “tertiary” cell wall.

Indeed, secondary cell walls are always made of distinct layers. When a xylem fiber develops a G-layer, it develops it before the completion of the regular secondary cell wall: the G-layer replaces the S3 and part or all of the S2 layers (Saiki, 1971; Abedini et al., 2015). The differentiation of G-fibers occurs by a modification in the sequence of secondary layers deposition, rather than adding a third phase to the cell wall development. It is even more obvious in the G-fibers of the S1 + G type (without S2 layer). Higaki et al. (2017) have shown that, in this kind of G-fiber, the G-layer presents a smooth variation in organization and composition from the outer to the inner side. The outer part of the G-layer is characterized by a large microfibril angle, the presence of xylan and lignification, very similar to the composition of the S2 layer. The inner part exhibits the more typical G-layer characteristics: low microfibril angle, less xylan, and lignification. Moreover, this study emphasizes the continuity between S to G type in a single layer. Other interesting cases are the tension wood fibers (Ruelle et al., 2007; Ghislain et al., 2016) and phloem fibers (Nakagawa et al., 2012, 2014) of some angiosperm families exhibiting multi-layered cell walls. These multi-layered fibers result from alternating G-layers and S3 layers. The G-layer may not be called tertiary if it is formed before a S3 and again appears to be one of the successive layers of the secondary cell wall.

Each step in a given developmental process is governed by a whole battery of specific transcription factors. However, the cell is also able to respond to environmental cues thanks to the mobilization of some transcription factors. Specific transcription factors have been shown to be upregulated in spruce compression wood (Bedon et al., 2007). These transcription factors (MYB2, MYB4, MYB8) are likely to play a role in lignin metabolism. Likewise, a drought stress induces the expression of specific transcription factors, regulating a specific set of genes, resulting in the development of tissues with modified structure and properties (Fujita et al., 2005; Fichot et al., 2009). In tension wood, it is known that lignin metabolism is switched off when G-layers are deposited. In consequence, it is expected that the expression of related transcription factors is down-regulated. This is observed for example for MYB58 and MYB63 (Gorshkov et al., 2017), which are transcriptional activators of the lignin biosynthetic pathway during secondary cell wall formation (Zhou et al., 2009). Therefore, the fact that some transcription factors appear differentially expressed between G-layer and S2 layer development is not sufficient to justify the differentiation of a tertiary layer.

## From a morphological view, is the G-layer a “tertiary” cell wall?

Based on the similarity between G-layers of poplar tension wood and flax phloem fibers, Gorshkova and collaborators point the fact that “tertiary cell walls have unique chemical and architectural features,” namely that they have cellulose microfibrils aligned with the cell axis, are thick, devoid of lignin and xylan, but contain rhamnogalacturonan-I. In fact, this definition of the G-layer seems too restrictive. Recent publications indicated that the G-layer presents a larger diversity of morphological features: G-layers can be very thin (Fang et al., 2007); G-layers may remain unlignified or become lignified according to the tree species (Roussel and Clair, 2015; Ghislain and Clair, 2017; Higaki et al., 2017); xylans have been evidenced in poplar G-layers (Guedes et al., 2017).

The G-layer is not the only cell wall layer with peculiar features. For example, a conifer compression wood tracheid exhibits a rounded shape that generates intercellular spaces, while its thick internal layer also has many typical traits: an exceptionally high microfibril angle (Timell, 1986), the presence of a specific hemicellulose (Wloch and Hejnowicz, 1983), a high lignin content with a typical GH-type composition (Nimz et al., 1981; Koutaniemi et al., 2007), the occurrence of important amounts of β 1-4 galactan (Altaner et al., 2007), lower mannose (Timell, 1967; Kartal and Lebow, 2001). It also has specific physical properties and is regulated by a specific set of transcription factors (Bedon et al., 2007; Villalobos et al., 2012). Nevertheless, this layer is identified as one of the secondary wall layers. Similarly, we think that the specificity of the G-layer may not justify classifying it apart from the other secondary wall layers. The historical term “G-layer” (as opposed to S-layer) already points clearly enough to its particular nature.

## How to define a G-layer?

Following the above discussion, we propose to dedicate the term tertiary cell wall to layers issued from a physiological process that fully differs from what happen in the secondary wall. Following Barnett and Jeronimidis (2003), we propose to name tertiary wall, what is built from a cell *post-mortem* process. This may include the warty layer as well as the late deposition of extractives (low molecular weight organic compounds, mainly terpenoids, alkaloids, or phenolic compounds, synthesized in living parenchyma cells) within the cell wall during heartwood formation.

We propose to define the G-layer as a part of the secondary wall, characterized by (i) an orientation of the cellulose microfibrils nearly parallel to the axis of the fiber, (ii) a matrix with high mesoporosity, and (iii) the ability to generate large contraction of the layer along the fiber axis during maturation, generating axial strain of the fiber or axial tensile stress if the strain is prevented. The G-layer may have microfibril angle in rupture with the surrounding S2 layer or be characterized by a continuous change in microfibril angle from S1 layer to G-layer. When the change in microfibril angle is abrupt, the G-layer is often observed detached from the S2 layer on cross sections. This detachment originates from the release of the high tensile stress in the G-layer during sectioning that produces a large strain in the G-layer, much larger than within the S2 layer. G-layer may be unlignified, partially lignified or fully lignified. Before lignification, the mesoporous texture gives the G-layer its gelatinous appearance, which has given the G-layer its name for more than a century and a half.

## Author contributions

All authors contributed equally to this work. They all participated to build the argumentation and write the paper. They all gave their final approval for publication.

### Conflict of interest statement

The authors declare that the research was conducted in the absence of any commercial or financial relationships that could be construed as a potential conflict of interest.
